# Cartilage Delamination Flap Mimicking a Torn Medial Meniscus

**DOI:** 10.1155/2016/7062129

**Published:** 2016-12-13

**Authors:** Gan Zhi-Wei Jonathan, Hamid Rahmatullah Bin Abd Razak, Mitra Amit Kanta

**Affiliations:** Singapore General Hospital, Outram Road, Singapore 169608

## Abstract

We report a case of a chondral delamination lesion due to medial parapatellar plica friction syndrome involving the medial femoral condyle. This mimicked a torn medial meniscus in clinical and radiological presentation. Arthroscopy revealed a chondral delamination flap, which was debrided. Diagnosis of chondral lesions in the knee can be challenging. Clinical examination and MRI have good accuracy for diagnosis and should be used in tandem. Early diagnosis and treatment of chondral lesions are important to prevent progression to early osteoarthritis.

## 1. Introduction

We report a case of a chondral delamination lesion due to medial parapatellar plica friction syndrome involving the medial femoral condyle. This mimicked a torn medial meniscus in clinical and radiological presentation.

## 2. Case Report

A 39-year-old gentleman presented to us in clinic with a primary complaint of right knee pain for 3 years, on a background history of previous right medial meniscus tear 3 years priorly. The pain was localized to the posteromedial aspect of the knee and was worse when squatting, kneeling, or walking down the stairs. His regular sporting activities involved cycling, which did not cause significant discomfort. There was no history of specific injury or trauma to the knee and no effusion. He reported crepitus from the knee. He had no previous operations of the knee.

On examination, the posterior one-third of the medial joint line was tender. No synovial swelling or effusion was detected. There was a palpable click when performing the patella grinding test, suggestive of injury to the patella or a medial parapatellar plica. The range of motion of the knee was normal.

The patient reported having a previous magnetic resonance imaging (MRI) scan of the right knee approximately 3 years and 9 months prior to the consult, which showed an intrasubstance medial meniscus tear. The pain had been constant since then.

A repeat MRI scan of the knee was performed (using a GE Healthcare Optima MR430s 1.5T machine). The following sequences were performed and reviewed: Proton Density (PD) sequences in coronal, sagittal, and axial cuts, Proton Density (PD) Fast Spin Echo (FSE) sequence in coronal cuts, and T2-weighted Fast Spin Echo (FSE) with fat suppression (FS) in sagittal cuts. The scan was reported as showing a horizontal tear of the posterior horn of the medial meniscus with superior articular surface contact, extending into the posterior root attachment (Figures 3 and 4). The anterior cruciate ligament was intact.

**Figure 1:**
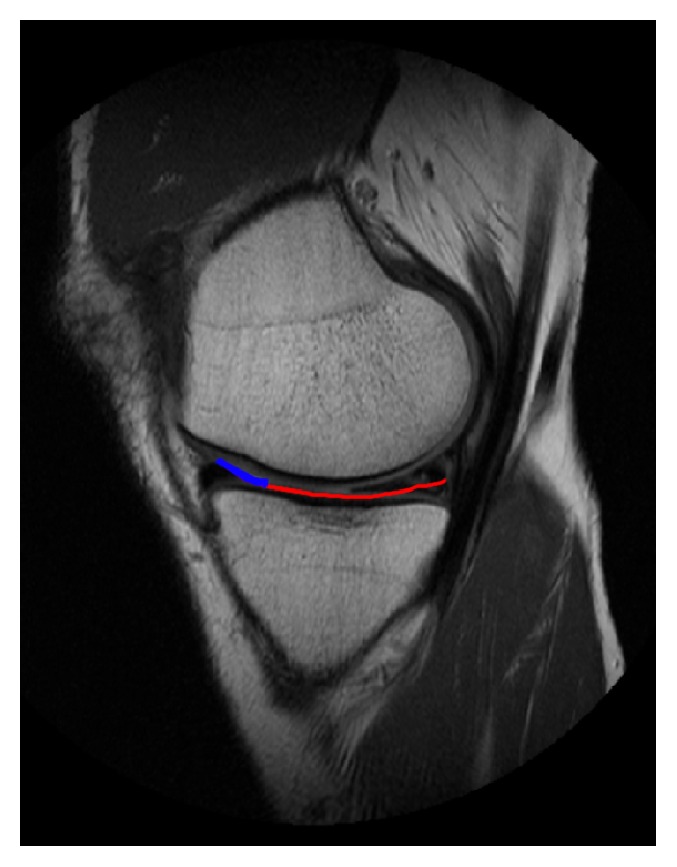
Cartilage flap indicated by red and blue line. The red line indicates the unattached, posterior two-thirds of the flap, and the blue line indicates the attached anterior third of the flap.

**Figure 2:**
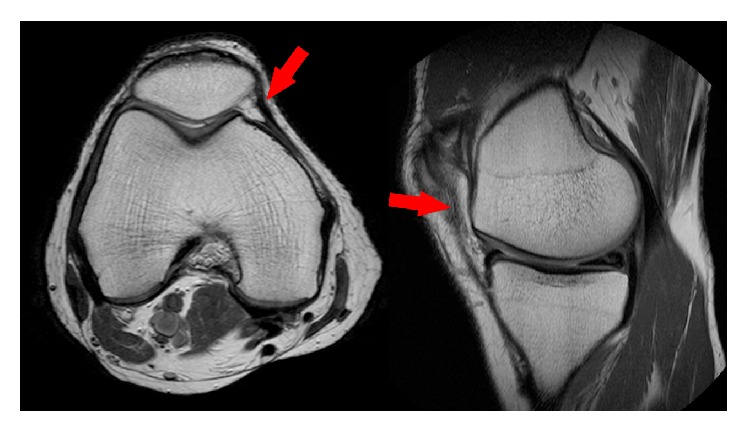
Prominent medial parapatellar plica indicated by arrowheads.

**Figure 3:**
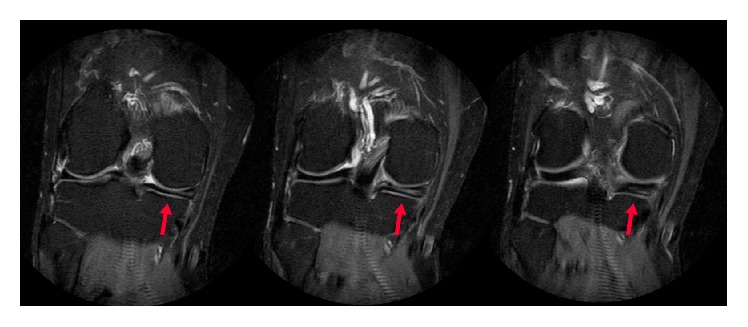
Coronal Proton Density (PD) fat suppression (FS) magnetic resonance imaging showing the chondral flap with an appearance similar to that of a torn medial meniscus (arrow). No underlying bone edema is seen.

**Figure 4:**
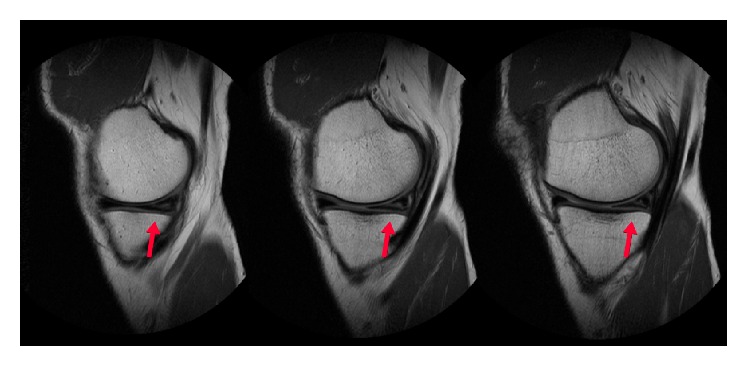
Sagittal Proton Density (PD) magnetic resonance imaging showing the chondral flap (arrow). It appears flap-like and is attached at its anterior aspect.

**Figure 5:**
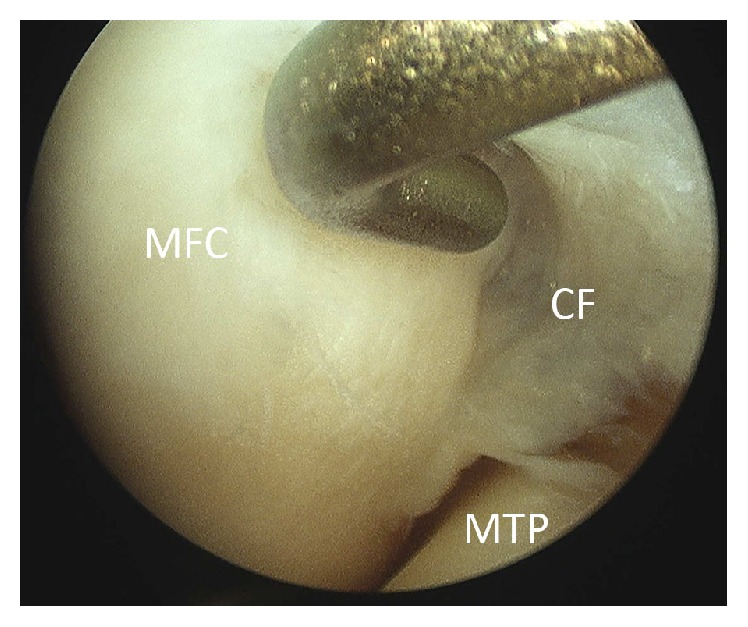
Arthroscopic view of the chondral flap. MFC: medial femoral condyle. CF: chondral flap. MTP: medial tibial plateau.

**Figure 6:**
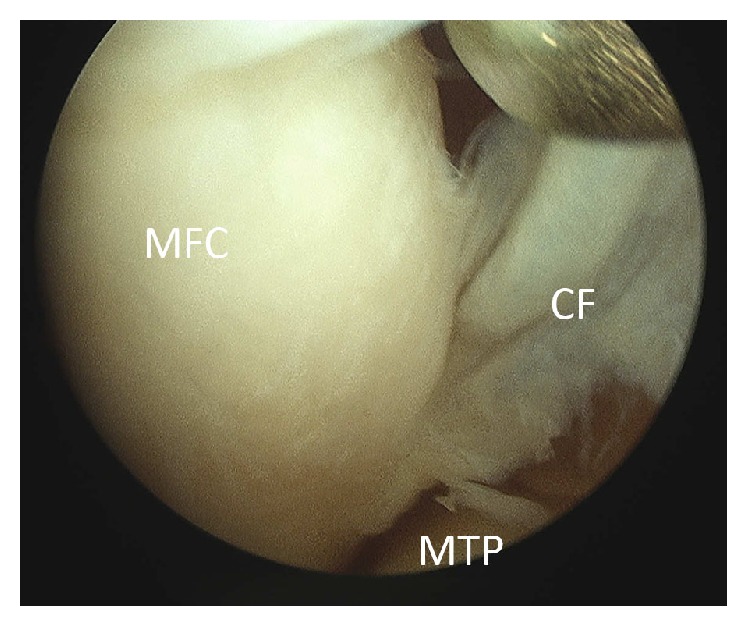
Arthroscopic view of the chondral flap. MFC: medial femoral condyle. CF: chondral flap. MTP: medial tibial plateau.

The patient underwent arthroscopy for treatment of the symptoms. During arthroscopy, a stiff medial parapatellar plica was noted, which was contacting and impinging on the medial femoral condyle (MFC) during knee flexion (Figures 5 and 6). Outerbridge grade 3 changes were noted of the cartilage in this area. A 2 × 2 cm cartilage flap was noted, attached anteriorly to the MFC (Figures 3 and 4). The flap was circular, approximately 2-3 mm thick, and attached along its anterior third to the anterior part of the medial femoral condyle (Figure 1). Its posterior two-thirds were free. There was no medial meniscus tear. Although not reported as showing a thickened medial parapatellar plica, review of the MRI showed a prominent medial parapatellar plica (Figure 2).

The lesion was debrided using a shaver until the remaining cartilage was stable with no loose edges. The cartilage underlying the flap showed Outerbridge grade 3 changes. The medial parapatellar plica was debrided, and no further impingement was noted during subsequent flexion/extension.

The patient's symptoms greatly improved after the operation. He was discharged the day after the operation, attended outpatient physiotherapy, and was able to resume normal work and activities after 2 week. He was sent for physiotherapy. During review at 3 months postoperatively, he was noted to have residual anterior knee pain, with some pain when squatting. Subsequently, during review 7 months after operation, the knee pain had resolved, with minimal pain when squatting, and he noted that his knee felt normal and he had recovered his strength. Knee examination was unremarkable.

## 3. Discussion

### 3.1. Common Clinical Presentation of Meniscal and Articular Cartilage Injuries

Meniscal injuries may present with pain, locking, catching, giving way, or pain when kneeling or squatting. Clinically, an effusion may be present. The Thessaly test, Mcmurray's test, and Apley's test may be positive, and joint line tenderness may be present. However, these tests have limited diagnostic accuracy [1–3], and further investigations are often required, such as magnetic resonance imaging (MRI) studies.

Articular cartilage injuries may present with pain, swelling, and locking. A history of injury, such as that of acute trauma, or twisting, may be present [4, 5]. Symptoms mimic a meniscal tear [6], and diagnosis may be challenging.

Parapatellar plica syndrome may present as anterior knee pain after prolonged sitting or when using the stairs. Retropatellar pain or medial knee pain may also be present. Other nonspecific symptoms such as intermittent clicking or locking or swelling may also occur [7]. A symptomatic medial parapatellar plica may be palpable on examination, especially if significantly thickened. During knee flexion between 30° and 60°, a snap or pop may be present. Palpation may also reveal retropatellar pain, clicking, or crepitations [8].

### 3.2. Structure of Cartilage

The structure of articular hyaline cartilage can be said to contain two large zones, a calcified and noncalcified zone. The noncalcified zone may be further subdivided into a superficial zone of thickness, in which collagen fibres are arranged parallel to the surface and offer good resistance to shear force, a transitional zone, in which collagen fibres run obliquely, and a deep zone, where collagen fibres are oriented perpendicularly to the surface and resist compression well. The calcified zone of cartilage contains cartilage fibres, anchored by hydroxyapatite crystals to the subchondral bone. The junction between the calcified and noncalcified zone is the tidemark [4, 9].

### 3.3. Clinical Presentation and Investigation of Injury

Partial-thickness separation or delamination injuries of articular cartilage similar to the one observed in our case (with the formation of a cartilage flap attached at one edge) have previously been described in the literature [5, 10, 11]. The delamination typically occurs at the tidemark, with the calcified zone of cartilage remaining attached to the subchondral bone.

The cartilage delamination in our patient was likely due to repeated injury and impingement from the stiff medial parapatellar plica; increasing Young's modulus of the plica is associated with greater contact pressures on the underlying cartilage [12]. Synovial plicae may cause injuries to the underlying cartilage through a combination of compression, friction, and shear forces [13] and are associated with an increase in underlying articular cartilage lesions when present in a joint [7, 14, 15].

In our case, the history and physical examination suggested a meniscal tear. The previous MRI scan findings of a torn medial meniscus in the context of pain since the time of diagnosis pointed to a torn medial meniscus as the cause of pain. This appeared to be borne out by the current MRI, which showed what we expected to see: a tear of the posterior horn of the medial meniscus.

History and clinical examination are an important step in the diagnosis of knee injuries. On its own, clinical examination can diagnose meniscal lesions with significant accuracy. Mohan et al. reported diagnostic accuracy of 88% for medial meniscus injuries and 92% for lateral meniscus injuries when compared to arthroscopy [16].

Clinical examination for meniscal injury has diagnostic accuracy similar to that of MRI [17–19] and when performed by an experienced surgeon may even surpass MRI [20]. Although other authors have noted less success (Sharma et al. found clinical accuracy of 73–78% compared to MRI accuracy of 92–95%, [21]); on the whole the accuracy of clinical examination remains high and should not be neglected in favour of MRI.

MRI is a good choice of imaging modality and has good sensitivity and specificity for diagnosis of meniscus tears. Sensitivities and specificities of over 80% have been described for detection of meniscal tears when compared with arthroscopy as a gold standard [22–24].

In contrast, MRI sensitivity for detection of articular cartilage injury is significantly lower than that for meniscal injury. A meta-analysis by Zhang et al. in 2013 found that sensitivity for detection of chondral injury was 75% (62%–84%) and overall specificity was 94% (89%–97%).

MRI features of chondral delamination after acute injury were described by Kendell et al. [25], who reported that all 5 of their cases showed increased T2-weighted (fast spin-echo) signal in subchondral bone underlying the cartilage injury, indicating oedema. Other authors have also described similar findings [26, 27].

It can be difficult to determine the exact Outerbridge grade of the chondral lesion on MRI. In addition, MRI has higher sensitivity for more severe lesions (Outerbridge grades 3 and 4), with a progressive decrease in sensitivity with lower Outerbridge grades [28]. Low-grade early lesions of the articular cartilage are less likely to be detected.

In our case, atypically, there was minimal subchondral oedema underlying the chondral flap, possibly due to a long interval between injury and diagnosis and the mechanism of injury. The mechanism of injury was likely to have been nontraumatic in nature or as a result of repetitive microtrauma (i.e., friction and/or shear force resulting from medial plica syndrome) rather than a typical cause of chondral injury (such as acute trauma or twisting injury). These factors might have contributed to the unusual features of the lesion.

Location of the lesion in the posterior aspect of the knee may have been another contributing factor. Imaging at or around the posterior meniscal horn can be challenging. Sharifah et al. described significantly lower sensitivities when the meniscal tears were located in the posterior horn [22]. In the same vein, Naranje et al. reviewed the accuracy of MRI for diagnosis of meniscal lesions [23]. Four out of 6 of their false-positive meniscal tears were in the posterior horn, which the authors felt could have been related to complex anatomy in this area.

MRI is a useful tool for diagnosis of plicae in the knee. Nakanishi et al. found the sensitivity of MRI (when compared to arthroscopy) to be 93.1% and specificity to be 81.8% [29]. Plicae have low intensity on T1-weighted and T2-weighted MR sequences. Presence of a knee effusion may improve visualization of plicae on MR imaging sequences [30].

### 3.4. Areas for Improvement of Diagnostic Accuracy with Magnetic Resonance Imaging: Magnet Strength

Identification and characterization of lesions may improve with use of a 3T magnet instead of a 1.5T magnet. The MRI in our study used a 1.5T magnet. Van dyck and Kenis et al. showed that sensitivity of detection of all grades of cartilage lesions in the knee joint improved with use of a 3T magnet [31].

### 3.5. Areas for Improvement of Diagnostic Accuracy with Magnetic Resonance Imaging: Use of Specific Sequences

Use of specific MR imaging sequences may improve diagnosis of articular cartilage lesions. Gustas et al. [32] reported in 2015 that use of a 3D FSE sequence with use of both radial and conventional reformatted images had improved sensitivity and similar specificity to use of 2D FSE sequences alone. Similarly, Kijowski et al. reported improvements in sensitivity with a small reduction in specificity with addition of a T2 mapping sequence when a 3T magnet was used [33]. Kohl et al. reported good results for Outerbridge grade III and IV lesions with a 3T magnet and 3D-DESS cartilage specific sequences [28].

### 3.6. Importance of Early Recognition of Articular Cartilage Injury

Early diagnosis of injury to the articular cartilage is important, because undiagnosed lesions represent an opportunity for further cartilage injury and early osteoarthritis. In particular, lesions larger than 9 mm result in increased pressure on the rims of the defects and will likely result in further chondrocyte insult and progression of cartilage injury [34]. The size of the defect in our case was approximately 20 mm by 20 mm and as such would likely see progressive worsening.

## 4. Conclusion

Pain from intra-articular knee injury may result from injury to various structures in the knee, including articular cartilage and menisci. Clinical presentation of chondral injury and meniscal injury may present similarly, and in some cases, accurate diagnosis may be challenging.

We recommend a focused history and clinical examination for complaints of knee pain, followed by magnetic resonance imaging with a 3T magnet if available, with relevant specific MR imaging sequences. Atypical cases such as ours are rare, but we should remain on high alert for chondral injury, as timely diagnosis and expeditious treatment may prevent worsening of defects and progression to early osteoarthritis.
